# Effects of Chickpea Protein on Carbohydrate Reactivity in Acrylamide Formation in Low Humidity Model Systems

**DOI:** 10.3390/foods9020167

**Published:** 2020-02-10

**Authors:** Karolina Miśkiewicz, Justyna Rosicka-Kaczmarek, Ewa Nebesny

**Affiliations:** Institute of Food Technology and Analysis, Faculty of Biotechnology and Food Sciences, Lodz University of Technology, Stefanowskiego Street 4/10 90-924 Lodz, Poland; karolina.miskiewicz@p.lodz.pl (K.M.); ewa.nebesny@p.lodz.pl (E.N.)

**Keywords:** acrylamide, low humidity model systems, asparagine, carbohydrates, chickpea protein isolate, differential scanning calorimetry, melting point

## Abstract

Asparagine and reducing sugars are the principal precursors of acrylamide in foods. Their main sources in pastries are flour and hen egg yolks. One method of reducing acrylamide content in food may be to add a chickpea protein preparation. The aim of the study was to determine the effects of the chickpea protein preparation on the thermodynamic properties of carbohydrates and the amount of acrylamide formed in low humidity model systems. In the studied systems, the type and amount of acrylamide precursors and humidity were designed to reflect the parameters typical of shortcrust cookies. In the study, the highest amounts of acrylamide were formed in the reaction between asparagine and fructose and the lowest in the reaction between asparagine and sucrose. Furthermore, the addition of chickpea protein to the analyzed carbohydrate–asparagine model systems reduced the content of acrylamide formed during baking at 180 °C regardless of the type of carbohydrate. The greatest acrylamide reduction (41%) was found in the model system containing fructose.

## 1. Introduction

Health properties are an essential attribute of any food product, which is especially true in the context of emerging reports on the hazards to human health and life associated with the discovery of acrylamide in food products subjected to thermal treatment [1]. According to nutritionists and dieticians, health quality is particularly important in the case of foodstuffs consumed widely and regularly for the duration of the entire life. These may certainly include shortcrust cookies, as cookies are the third largest segment of the global confectionery market. Over recent years, their worldwide output has been on the increase, reaching a value of over 63 billion euros in 2013.

Shortcrust cookies belong to the group of food products that can be classified as convenience foods, enjoying great popularity among consumers [2]. Their appeal is enhanced by their low price, relatively high nutritional value, availability of different flavors, and relatively long shelf life. The main ingredients of shortcrust cookies are flour, egg yolk, and sucrose, which are sources of carbohydrates and amino acids, including precursors for acrylamide formation [3]. Heat treatment (baking) is a critical step in the production of shortcrust cookies, leading to their specific taste and flavor. Given a baking temperature of more than 120°C and the composition of ingredients, shortcrust cookies are a potential source of harmful substances, such as acrylamide and its derivatives [4]. According to the literature data, the content of acrylamide in shortcrust cookies ranges between 30 µg kg^−1^ and 3200 µg kg^−1^ [5].

Acrylamide is formed in the Maillard reaction, when the compounds responsible for the taste and smell of the golden-brown crust on the products are generated. One hypothesis suggests that acrylamide is generated via reactions between reducing sugars, primarily glucose and fructose, and amino acids, mainly asparagine, which are present in large quantities, e.g., in cereal products.

Acrylamide as a potential carcinogen for humans may contribute to many diseases, including those with a genetic component [6]. Consequently, it is necessary to seek effective methods of reducing its content in food. One of such methods involves the addition of a chickpea protein preparation. Research conducted by Vattem and Shetty [7] showed that adding chickpea protein to potato slices prior to frying significantly reduced the amount of acrylamide present in the finished product.

The literature data indicate that the type of carbohydrate or carbonyl compound present in the reaction environment may have a significant impact on the amount of acrylamide generated in the Maillard reaction [8,9]. Some authors have investigated the formation of acrylamide in carbohydrate reactions with amino acids in model systems. However, those systems involved equimolar amounts of carbohydrate and asparagine, and the reactions were typically conducted in aqueous media with a phosphate buffer of suitable pH [10] or water [11]. It should be emphasized, however, that those model systems did not include chickpea protein. Indeed, in the available literature there is no information on the impact of chickpea protein in selected confectionery products on acrylamide concentration, which is to say that there are no data on the thermal stability of reducing sugars and sucrose in the presence of chickpea protein in model systems reflecting the typical composition of shortcrust cookies.

The aim of the present study was to determine the effects of a chickpea protein preparation on the thermodynamic properties of carbohydrates and the amount of acrylamide formed in low humidity model systems. Those systems consisted of glucose, fructose, sucrose, and asparagine, which are precursors for acrylamide formation, in amounts at which they occur in the main ingredients of shortcrust pastries (wheat flour, egg yolk, and table sugar). In addition, water content in the tested systems was approx. 15% to ensure that their consistency would have a structure resembling that of shortcrust cookie dough. Furthermore, a chickpea protein preparation was added to selected systems.

The parameters of model systems used, i.e., the concentration of precursors and moisture content, have not been previously studied in this perspective [10,11]. Moreover, the existing literature has not addressed the presence of a factor limiting the formation of acrylamide in the form of a chickpea protein preparation under the above conditions.

## 2. Materials and Methods

### 2.1. Chemicals

Asparagine (99%) was purchased from Sigma-Aldrich, carbohydrates (>98%) (glucose, fructose, sucrose) from Fluka, acrylamide (>99%) from Merck (Darmstadt, Germany), 2,3,3-d3-acrylamide (>98%) from Cambridge Isotope Laboratories, Inc. (Andover, MA, USA), from Sigma- Aldrich (St. Louis, MO, USA), ethyl acetate and methanol (both of HPLC-grade) from J. T. Baker (Deventer, The Netherlands), and HPLC-grade acetonitrile from Scharlab (Barcelona, Spain). Hexane (99%), Br (99.5%), anhydrous sodium acetate (>99%), bromine water, formic acid, Carrez I, and Carrez II were from Chempur (Piekary Śląskie, Poland). Ultra-purified water (resistivity 18.2 MΩ cm^−1^) was obtained using Milli-Q Plus Technology, Millipore (Bedford, MA, USA). C 18-E (55μm, 70Å, 6 mL, 500 mg) SPE cartridges were purchased from Shim-Pol (Izabelin, Poland).

### 2.2. Making Chickpea Protein Preparation

An aqueous suspension of previously defatted chickpea flour was prepared in the ratio 1:10 (*w*/*v*). The pH of the suspension was adjusted to 8.3 using 0.2% NaOH. Protein was extracted from the solution by shaking the suspension for 60 min at 30 °C and centrifugation under the following conditions: 3024× *g*, 20 min, 4 °C. The supernatant was decanted and the dry residue was used for subsequent extraction under the same conditions but using half of the solvent. The pH of the combined supernatants was brought to 4.3 (the isoelectric point of chickpea protein) using 1N HCl. The protein obtained under these conditions was centrifuged. The protein precipitate was washed with distilled water at pH 4. 3 and then centrifuged (3024× *g*, 20 min, 4 °C). The obtained protein residue was freeze-dried.

### 2.3. Preparation of the Model Systems

In the model systems, reducing sugars, i.e., glucose and fructose, as well as asparagine were present in amounts similar to those in wheat flour (fructose—1.03 mg g^−1^ d.m., glucose—2.19 mg g^−1^ d.m., asparagine—5.21 mg 100 g^−1^ d.m.) and egg yolk (glucose—0.25 mg g^−1^ d.m., asparagine—1.90 mg 100 g^−1^ d.m.), which are the ingredients of shortcrust pastries (Table 1). The content of sucrose in model systems was the sum of its content in flour (5.15 mg g^−1^ d.m.) and egg yolk (0.01 mg g^−1^ d.m.) as well as the amount of sucrose added according to the recipe in the form of table sugar (25.00 g 100 g^−1^, see Table 1). The ingredients of shortcrust pastries were used at a ratio of 0.5/0.3/0.16 (*w*/*w*) for wheat flour, fat, and table sugar, respectively. The chickpea protein preparation used in experiments had the following composition: protein—82.70 g 100 g^−1^ d.m., fructose—0.05 mg g^−1^ d.m., glucose—0.02 mg g^−1^ d.m., sucrose—0.12 mg g^−1^ d.m., and maltose—1.49 mg g^−1^ d.m. Chickpea protein was added to the model systems at the expense of the filler, i.e., silicon dioxide. This compound is devoid of taste and smell.

The use of silicon dioxide ensured adequate moisture of the model systems. The moisture and pH of the systems corresponded to those of shortcrust cookie dough. The moisture content of the model systems was 14.84%, as adjusted by the addition of an appropriate amount of water, taking into consideration the amount of water introduced with the other ingredients. The pH was 6.68.

The model systems were prepared by combining all the ingredients according to the recipes shown in Table 1, in an appropriate order. The model systems were shaped like cookies (45 mm in diameter, 3 mm thick), placed on a baking sheet, and baked in dry air (relative humidity 0.3%) at 180°C for 8 min. Then, they were cooled to 25 °C and stored at –20 °C until analysis.

### 2.4. Analyses of Model Systems

#### 2.4.1. Amino Acid Content

The content of free amino acids in model systems was determined according to the Commission Directive 98/64/EC [12]. First, 10 mL of an extraction mixture composed of 0.1 mol L^−1^ HCl with 2% thiodiglycol (2,2’-thiodiethanol, TDE) was added to 1 g of a ground sample (weighted with an accuracy of 0.0001 g). Extraction was carried out in a shaking water bath for 60 min at room temperature. After reaction completion, the samples were centrifuged (3024× *g*, 10 min, 20 °C) and then defatted twice with hexane with centrifugation after each defatting (3024× *g*, 10 min, 10 °C). Subsequently, 2 mL of a defatted sample was collected into a glass vial, 1 mL of sulfosalicylic acid was added with continuous stirring (using a magnetic stirrer), and the whole was stirred for another 5 min. After that time, the solution was transferred to plastic tubes and centrifuged (3024× *g*, 5 min, 20 °C). The supernatant was collected and treated with 2 mL of pH 2.2 buffer. Then, the sample was filtered through a nylon membrane filter with 0.2 μm pore size to autosampler vials and subjected to chromatographic analysis.

The separation and quantitative analysis of free amino acids were carried out using a Biochrom 30+ amino acid analyzer. The identification of separated amino acids was done by comparing the retention times of chromatographic fractions from the samples with standard mixtures. The free amino acids present in the analyzed material were quantified by comparing their peak areas with the peak areas of the corresponding standards.

#### 2.4.2. Carbohydrate Content by HPLC

The content of carbohydrates in model systems was determined on Shodex NH2P-50 series columns, as previously reported [13], with some modifications. First, 2 g of a sample was weighed into a 50 mL centrifuge tube with an accuracy of 0.0001 g, 15 mL of ultrapure water was added, and the tube was inserted into a shaking water bath at 58 °C for 45 min. The sample was then centrifuged (3024× *g*, 20 min, 20 °C). After that, the supernatant was decanted and defatted twice with 10 mL of hexane. Defatted samples were then deproteinized by adding 0.1 mL of Carrez I and II reagents and centrifuging (3024× *g*, 10 min, 20 °C). After centrifugation, the supernatant was quickly decanted and filtered through a nylon syringe filter with 0.2 μm pore size and evaporated to dryness in a rotary evaporator at 40 °C (Büchi V-855, R-210/215 equipped with a V-700 vacuum pump). The residue from concentration was dissolved in 5 mL of ultrapure water. The sample was then subjected to extraction using SPE with C-18 extraction columns. Before extraction, the column was washed with 5 mL of HPLC-grade methanol and then with 5 mL of ultrapure distilled water. The column was conditioned at a low flow rate to avoid dehydration of the sorbent. Subsequently, the entire volume of the sample was introduced without the use of vacuum. Sugars were eluted with 1 mL of ultrapure distilled water. The samples prepared in this way were filtered through a 0.45 μm pore size syringe filter. The filtrate was analyzed for the content of free sugars using a UHPLC+ Dionex UltiMate 3000 system (Thermo Fisher Scientific Inc., Waltham, MA, USA) equipped with a refractive index detector (Shimadzu, Kioto, Japan)and an Asahipak NH2P-50 4E column (4.6 × 150 mm, 5.0 μm particle size; Shodex, Japan). Isocratic elution was carried out with 70/30 (*v*/*v*) acetonitrile/water as the mobile phase. The flow rate was set to 1.0 mL/min and column temperature was 30 °C. Glucose, sucrose, and fructose were identified by comparing their retention times with authentic standards. Quantification was done using an external standard method.

#### 2.4.3. Acrylamide Content Determination by Gas Chromatography-Mass Spectrometry with Derivatization

Acrylamide content was calculated on the basis of the determination of acrylamide dibromo derivatives by gas chromatography–mass spectrometry (GC-MS/MS). Acrylamide content in model systems subjected to baking was determined according to Alves [14], Mojska [15] and Soares [16].

First, 2 g of a ground sample was weighed into a 50 mL centrifuge tube with an accuracy of 0.0001 g, followed by the addition of 100 µL of 100 µM deuterated acrylamide standard solution and 20 mL of double-distilled water. The capped tube was placed in a shaking water bath at 60°C and extraction was carried out for 30 min. After cooling to room temperature, the extract was centrifuged (3024× *g*, 20 min, 4 °C). After separation from the precipitate, the supernatant was defatted three times with decreasing volumes of hexane. For this purpose, 20 mL, 15 mL, and 10 mL of hexane were added to the supernatant, respectively, and the whole was vortexed for 1 min and centrifuged (3024× *g*, 10 min, 10 °C). The upper hexane layer was decanted. To the lower aqueous layer, 0.3 mL of Carrez solution I and II was added for deproteinization. The whole was then slightly shaken and centrifuged (3024× *g*, 10 min, 10 °C). The obtained extract was subjected to overnight bromination. For this purpose, 2.5 g of KBr, 0.1 mL of HBr at pH ~ 1–3 and 2.5 mL of bromine water was introduced to the cold extract. Bromination was carried out in a water bath at approx. 0 °C, in the dark. Excess bromine in the solution was decomposed by adding a few drops of 1 M sodium thiosulfate. The disappearance of the yellow color indicated the completion of the reaction. Then, 4 g of NaCl was added to the mixture and dissolved. The mixture was extracted twice for 5 min with 4 mL of ethyl acetate. After decantation, the ethyl acetate layer was collected and approx. 4 g of anhydrous sodium sulfate was introduced to dry the extract, which was followed by centrifugation (3024× *g*, 10 min, 4 °C). The organic layer of the extract was evaporated to dryness under a stream of nitrogen at 40 °C. After evaporation, 200 μL of ethyl acetate was added and transferred into 2 mL dark autosampler vials.

The analysis parameters were selected according to Miśkiewicz et al. [3]. The 2,3-dibromo derivative of acrylamide was quantified by GC using a Varian 450-GC gas chromatograph equipped with an ion trap mass detector (Varian 220-MS) and a split/splitless injector. Analytical separation was performed on a Varian Factor Four VF-5ms capillary column (0.25 µm film thickness, 30 m 0.25 mm i.d.). Samples (1 µL) were analyzed at an ionization energy (EI) of 70 eV. In the first step, precursor ions with *m*/*z* of 152 and 155 were obtained from the 2,3-dibromo acrylamide derivative and the 2,3-dibromo derivative of deuterated acrylamide, respectively. Their collisions gave rise to daughter ions with *m*/*z* of 135 (from ions with *m*/*z* of 152) and 137 (from ions with *m*/*z* of 155). Calculations of acrylamide concentration in the tested samples were based on the ratio of surface areas under peaks corresponding to ions with *m*/*z* of 135 and 137.

Parameters of gas chromatography:

-Temperature increase from 65 °C to 240 °C over 23 min (at a rate of 15 °C min^−1^);

-Injector temperature: 250 °C;

-Carrier gas: helium at a flow rate of 40 mL s^-1^.

Parameters of mass spectrometry:

-Ionization energy: 70 eV;

-Ion source temperature: 180 °C;

-Transfer line temperature: 250 °C.

Quantification was performed by the internal standard method described by Miśkiewicz et al. [3]. A calibration curve was constructed by plotting the Aaa/Ais ratio against Caa/Cis, where Aaa is the area of unlabeled acrylamide with mass trace *m*/*z* of 135, and Ais is the area of deuterium-labeled acrylamide with mass trace of *m*/*z* 137. Caa/Cis designates the concentration ratio of acrylamide and 2,3,3-d3-acrylamide.

A calibration line was prepared in the range of 2.5–500 µg L^−1^. The correlation coefficients were usually higher than 0.998. The limits of detection (LOD) and quantification (LOQ) were calculated using the calibration curve parameters to be 5.0 μg kg^−1^ and 15.1 μg kg^−1^, respectively. Recovery was measured by adding 50 µg L^−1^ of the acrylamide standard solution to the sample. Average recoveries ranged from 73% to 89%.

#### 2.4.4. Thermal Analysis of Reducing Sugars and Sucrose by Differential Scanning Calorimetry (DSC)

Reducing sugars and sucrose in the model systems were subjected to thermal analysis using the DSC method according to Robert et al. [17] and Hurtta et al. [18]. Measurements were performed by means of a Setaram DSC 111 differential calorimeter (France). A 15 mg sample of an appropriate carbohydrate was placed in a measuring cell (“batch” type steel crucible) and heated from 25 °C to 210 °C with a scanning (heating) speed of 3 °C min^−1^. The applied temperature range enabled the observation of thermal changes in the analyzed sugars, depending on their type. An empty cell served as a reference. During the heating step, changes in sugar polymorphism were recorded in the form of peaks in endothermic curves. The value of enthalpy change Δ*H* expressed in J g^−1^ of the sample was calculated from the surface area under the peaks. Temperature was determined in °C at the beginning of the melting process (*T*_onset_) and at the end of the melting process (*T*_offset_); in addition, the peak temperature was recorded (*T*_max_). Thermal analysis was used to determine the effects of carbohydrate type and chickpea protein addition on the thermodynamic properties of carbohydrates and on the amount of acrylamide formed.

### 2.5. Statistical Analysis

All analyses were carried out in triplicate with the results expressed as means and standard deviations (SD). The significance of differences was determined using Tukey’s *t*-test. Results at a significance level *p* < 0.05 were accepted as statistically significant. Statistical evaluation is shown in tables and figures, with statistically different results being labeled with different letters.

## 3. Results and Discussion

The aim of the study was to determine the effects of the addition of a chickpea protein preparation on the thermodynamic properties of the analyzed carbohydrates and the amount of acrylamide formed in low humidity model systems.

### 3.1. Effects of Baking Conditions and Chickpea Protein Preparation on Asparagine and Carbohydrate Content in Low Humidity Model systems

**Figure 1:**
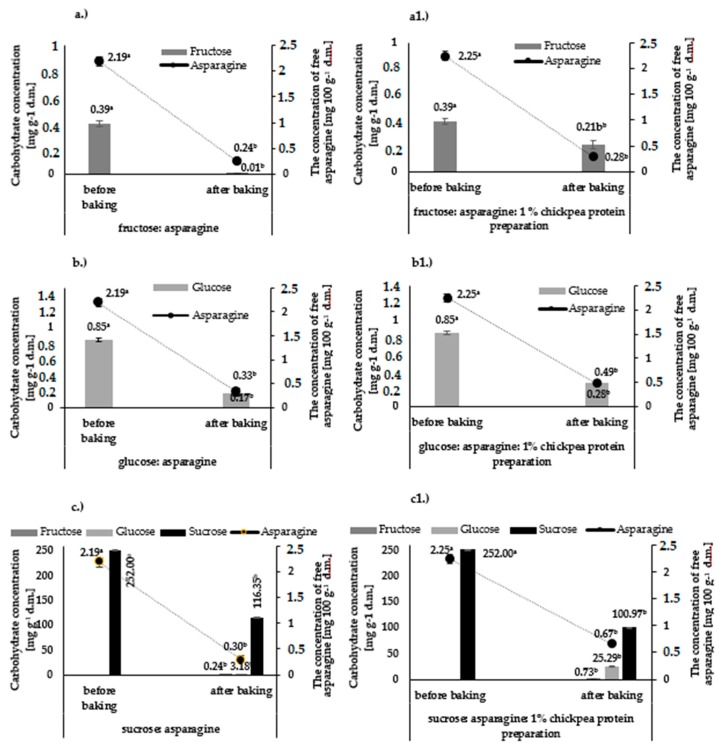
Concentration of carbohydrates and free asparagine in model systems with and without 1% chickpea protein preparation: fructose: asparagine (**a**), fructose–asparagine–1% chickpea protein preparation (**a1**), glucose–asparagine (**b**), glucose–asparagine–1% chickpea protein preparation (**b1**), sucrose–asparagine (**c**), sucrose–asparagine–1% chickpea protein preparation (**c1**)**,** before and after baking; lowercase letters—different letters in the same figure indicate significant differences in the content of individual carbohydrates and asparagine within the same model system before and after baking (*n* = 3; *p* ≤ 0.05); data are presented as means ± SD.

Asparagine is a necessary substrate in the acrylamide formation reaction. The content of asparagine in the model systems not subjected to thermal treatment was 2.19 mg 100 g^−1^ d.m. without chickpea protein (Figure 1a–c) and 2.25 mg 100 g^−1^ d.m. in systems with the addition of 1% chickpea protein (Figure 1a1–c1).

The process of baking at 180 °C for 8 min significantly reduced the content of free asparagine as compared to its initial concentration regardless of the carbohydrate used or the presence of chickpea protein preparation (Figure 1a–c,a1–c1). The content of free asparagine after baking ranged from 0.24 mg 100 g^−1^ d.m. for the fructose–asparagine system (Figure 1a1) to 0.67 mg 100 g^−1^ d.m. for the asparagine–sucrose system with 1% chickpea protein preparation (Figure 1c1).

The highest reduction in asparagine content as compared to its initial concentration in unbaked model systems was found for fructose: asparagine (89.0%) and fructose–asparagine–1% chickpea protein preparation (87.5%) model systems. The lowest reduction in the asparagine content was found for the sucrose: asparagine (86.3%) and sucrose: asparagine: 1% chickpea protein (70.2%) model systems (Table 2).

The following carbohydrates were used for the preparation of model systems: fructose in the amount of 0.39 mg g^−1^ of the system, glucose in the amount of 0.85 mg g^−1^, and sucrose in the amount of 252 mg g^−1^ (Figure 1a–c,a1–c1). The content of each reducing sugar in the tested carbohydrate–asparagine model systems corresponded to the sum of the sugars derived from wheat flour and egg yolk. The amount of sucrose was the sum of its content in wheat flour, egg yolk, and the amount of table sugar added according to the formulation for shortcrust pastries. Comparative analysis of carbohydrate content in model systems before and after baking showed a decrease of each of the analyzed carbohydrates upon thermal treatment. Moreover, the presence of glucose and fructose was detected in the sucrose: asparagine and sucrose: asparagine: 1% chickpea protein model systems, while they were absent in the raw model systems (Figure 1c1), so their presence is attributable to sucrose hydrolysis during the baking process. Comparative analysis also showed that the carbohydrate most reactive with asparagine was fructose, which reacted in 97.4%, while the least reactive compound was sucrose, which reacted only in 52.5% (Table 2).

The protein preparation used in analysis also contains some free asparagine (7.65 mg 100 g^−1^ d.m), and so its addition introduces an additional amount of asparagine to the carbohydrate–asparagine model systems. Nevertheless, despite that, a smaller decrease was observed in the concentration of asparagine and each of the carbohydrates during the baking process.

This may be explained by the protective activity of chickpea protein, which can form complexes with carbohydrates, thus reducing their availability for the reaction; proximity to asparagine renders complex formation necessary in the acrylamide formation reaction.

### 3.2. Effects of Reducing Sugar Type and Chickpea Protein Preparation on Acrylamide Content in Low Humidity Model Systems

**Figure 2:**
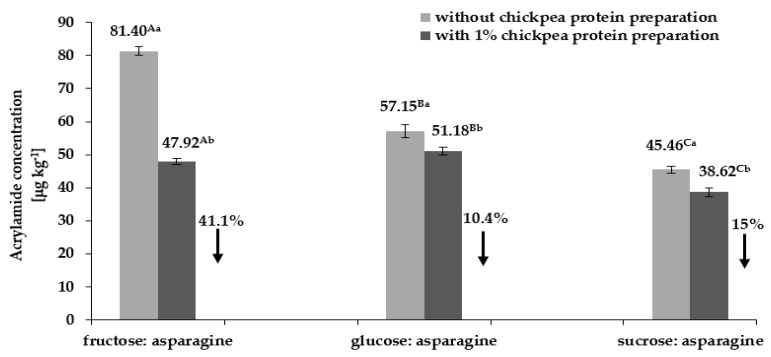
Changes in acrylamide concentration in carbohydrate–asparagine model systems with 1% chickpea protein preparation, subjected to thermal treatment, depending on carbohydrate type; uppercase letters—different letters indicate significant differences in acrylamide content between systems depending on carbohydrate type regardless of the addition of chickpea protein (*n* = 3; *p* ≤ 0.05); lowercase letters—different letters indicate significant differences in acrylamide content in a given model system depending on the addition of chickpea protein preparation (*n* = 3; *p* ≤ 0.05); data are presented as mean ± SD.

Acrylamide content in low humidity model systems containing two (carbohydrate–asparagine) and three (carbohydrate–asparagine–1% chickpea protein preparation) components is shown in Figure 2. The highest acrylamide content was found in the model system consisting of asparagine and fructose (81.40 µg kg^−1^), and the lowest in the system with asparagine, sucrose, and 1% chickpea protein preparation (38.62 µg kg^−1^). Acrylamide concentration in food products is known to depend on the presence of asparagine and reducing sugars [19], with one of the determinants being carbohydrate type.

The data presented in Figure 2 show the impact of carbohydrate type on the amount of acrylamide formed by heating carbohydrate–asparagine model systems. The replacement of fructose with glucose or sucrose in carbohydrate–asparagine model system caused a decrease in the resulting acrylamide content by 29.8% and 44.1%, respectively.

According to Yuan et al. [20], glucose, which is an aldose, is more reactive than fructose, a ketose, due to the presence of highly reactive aldehyde groups in aldose molecules. In the process of acrylamide formation, the situation is reversed with fructose being more reactive than glucose [19]. As indicated in the literature [17], this is due to the low melting point of the former carbohydrate. Gőkmen and Şenyuva [21] reported that the melting points of reagents have a major impact on acrylamide formation in reactions between asparagine and reducing sugars. Moreover, the melting point of a carbohydrate affects its reactivity with asparagine in acrylamide formation reactions.

There is an inverse correlation between the melting point of the carbohydrate and the degree of asparagine-to-acrylamide conversion. Carbohydrates used in the present study have the following melting points: fructose 119 °C–122 °C, glucose 152 °C, and sucrose 184 °C, for, glucose, and sucrose, respectively [17]. Due to its high melting point, sucrose is the least reactive among the analyzed carbohydrates and reacts with asparagine to a much lesser extent, which in turn leads to the formation of lesser amounts of acrylamide (Figure 2). On the other hand, fructose, having the lowest melting point, is the most reactive in low humidity model systems, and hence the highest amounts of acrylamide were formed as a result of its heating with asparagine (a high degree of asparagine-to-acrylamide conversion as seen from Figure 1a–c1 and Figure 2.). This is consistent with the data presented by Curtis et al. [22].

Analysis of the results also showed a significant effect of chickpea protein preparation on acrylamide content in carbohydrate–asparagine model systems. The data presented in Figure 2 show the reduction of acrylamide content in carbohydrate-asparagine–1% chickpea protein preparation model systems irrespective of the type of carbohydrate used. The highest reduction in acrylamide content (41.1%) was observed for the fructose–asparagine system, and the lowest (10.4%) for the glucose–asparagine system. This can be explained by the fact that in the former case fructose reacted with asparagine only in 46%, while in the latter case a much higher reduction in glucose content (67%) was observed as compared to its content in a model system without chickpea protein (Table 2).

### 3.3. Effects of Chickpea Protein Preparation on the Thermal Stability of Reducing Sugars

In order to confirm the reports of Robert et al. [17] and Curtis et al. [22] on the effects of the melting points of carbohydrates on acrylamide formation in carbohydrate–asparagine model systems, carbohydrates as well as mixtures of carbohydrates with the chickpea protein preparation were analyzed by DSC.

Table 3 shows the enthalpy of fusion of the analyzed carbohydrates and their melting points, i.e., the temperature at the start of the process (*T*_onset_), the temperature at the end of the process (*T*_offset_), and the difference between the two (*T*_offset_–*T*_onset_).

**Figure 3:**
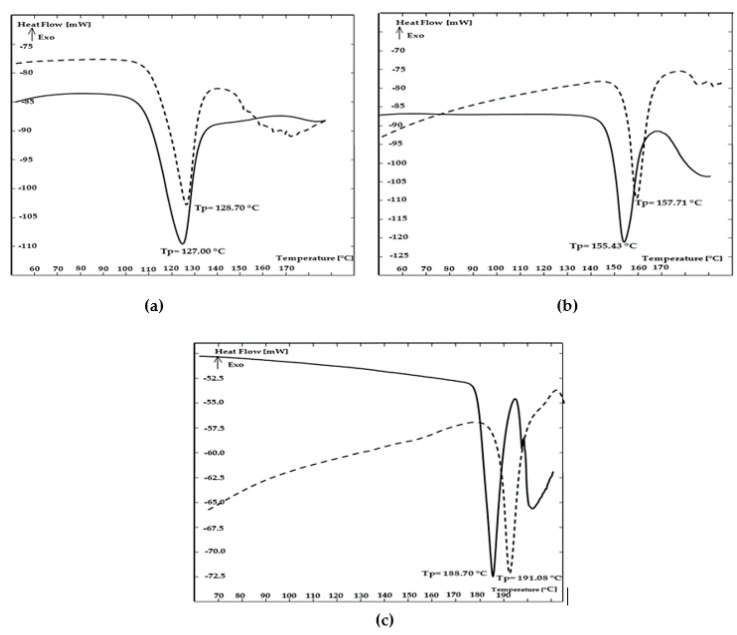
DSC thermograms for fructose (**a**), glucose (**b**), and sucrose (**c**), with and without the addition of chickpea protein preparation; solid line—sugar without chickpea protein; dotted line—sugar with chickpea protein.

On the basis of the endothermic curves shown in Figure 3a–c, the melting points of the studied carbohydrates were found to be *T*_max_ = 127.00 °C for fructose, *T*_max_ = 155.43 °C for glucose, and *T*_max_ = 188.70 °C for sucrose. The difference between the starting and end temperatures (*T*_offset_–*T*_onset_) indicates the degree of crystallinity of a given carbohydrate, and thus its uniformity and availability for asparagine in the acrylamide formation reaction. Comparative analysis of this parameter showed that fructose had the lowest degree of crystallinity (*T*_offset_–*T*_onset_ = 24.05 °C), and sucrose the highest degree (*T*_offset_–*T*_onset_ = 11.90 °C, see Table 3).

DSC results also confirm the effects of chickpea protein on the transformation of the analyzed carbohydrates, which is reflected in their higher melting points. The greatest increase in melting point was observed for sucrose (2.33 °C), and the lowest for fructose (1.69 °C, see Figure 3a–c). The obtained results show that regardless of carbohydrate type, the addition of chickpea protein reduced the difference between the temperatures of the melting process (*T*_offset_–*T*_onset_).

This indicates higher ordering of the crystallographic structures of the carbohydrates and thus their lower availability for asparagine in reactions leading to acrylamide formation.

As compared to carbohydrates without the addition of the preparation, the greatest reduction in the *T*_offset_–*T*_onset_ difference was observed for fructose, and the lowest for sucrose, which was reflected in the amount of acrylamide formed. This means that the addition of chickpea protein to carbohydrate–asparagine model systems resulted in the greatest reduction in acrylamide content for fructose and the lowest reduction for sucrose.

## 4. Conclusions

The present study showed a significant effect of carbohydrate type as well as chickpea protein preparation on the amount of acrylamide formed in low humidity model systems. In addition, the effect of chickpea protein on carbohydrate thermal stability was revealed, which determined their reactivity in terms of acrylamide formation.

Model systems accurately reflecting the composition of acrylamide precursors in shortcrust pastry dough, as well as imitating its humidity conditions, showed that the highest amounts of acrylamide were formed in the reaction between asparagine and fructose and the lowest in the reaction between asparagine and sucrose. Furthermore, it was found that the addition of a chickpea protein preparation to the analyzed carbohydrate–asparagine model systems reduced the content of acrylamide formed during heating at 180 °C regardless of carbohydrate type. The greatest acrylamide reduction (41%) was obtained in a model system with fructose. In contrast, in another study the use of rosemary extract in shortcrust cookies reduced their acrylamide content only by 15% to 18%, depending on air humidity during baking [23].

DSC results may suggest that among the analyzed carbohydrates fructose was characterized by the lowest thermal stability, i.e., the value of the difference *T*_offset_–*T*_onset_ indicate the lowest degree of crystallinity, which made it more reactive with asparagine. The addition of chickpea protein to carbohydrate–asparagine model systems led to increased crystallinity of carbohydrate structures. This shows the importance of the *T*_offset_–*T*_onset_ parameter, which reflects the degree of crystallinity of a given compound and determines, inter alia, the reactivity of carbohydrates with asparagine in acrylamide formation reactions. The obtained results clearly indicate that the applied chickpea protein preparation can be used to reduce the concentration of acrylamide in shortcrust cookies much more effectively than other known methods.

## Figures and Tables

**Table 1:** Qualitative and quantitative composition of amino acid: carbohydrate model systems.

Component	Fructose: Asparagine		Glucose: Asparagine		Sucrose: Asparagine	
	Without Chickpea Protein	With 1% Chickpea Protein	Without Chickpea Protein	With 1% Chickpea Protein	Without Chickpea Protein	With 1% Chickpea Protein
	[g 100 g^−1^ of the Product]					
Fructose	0.039	0.039	nd	nd	nd	nd
Glucose	nd	nd	0.085	0.085	nd	nd
Sucrose	nd	nd	nd	nd	25.20	25.20
Asparagine	0.0022	0.0022	0.0022	0.0022	0.0022	0.0022
Chickpea protein preparation	nd	0.37	nd	0.37	nd	0.37
Silicon dioxide	99.96	99.59	99.91	99.54	74.80	74.43
Water added to the system	9.99	9.96	9.99	9.95	7.48	7.44

nd—not detected.

**Table 2:** Changes in the content of monosaccharides, sucrose, and the amino acid asparagine in model systems with and without the addition of a chickpea protein preparation, subjected to baking.

	Glucose-Asparagine		Fructose-Asparagine		Sucrose-Asparagine	
	Without Chickpea Protein	With 1% Chickpea Protein	Without Chickpea Protein	With 1% Chickpea Protein	Without Chickpea Protein	With 1% Chickpea Protein
Fructose	nd	nd	97.4% reduction	46.0% reduction	0.24-fold increase	0.73-fold increase
Glucose	80.0% reduction	67.0% reduction	nd	nd	3.18-fold increase	25.29-fold increase
Sucrose	nd	nd	nd	nd	53.8% reduction	59.9% reduction
Asparagine	84.9% reduction	78.2% reduction	89.0% reduction	87.5 reduction	86.3% reduction	70.2% reduction

nd—not detected.

**Table 3:** Effects of chickpea protein preparation on thermal changes occurring in reducing sugars and sucrose during heating.

	Fructose		Glucose		Sucrose	
	Without Chickpea Protein	With 1% Chickpea Protein	Without Chickpea Protein	With 1% Chickpea Protein	Without Chickpea Protein	With 1% Chickpea Protein
*T*_onset_ [°C]	112.02 ± 0.95	116.46 ± 1.01	150.83 ± 0.76	152.95 ± 0.83	183.99 ± 0.97	186.44 ± 0.66
*T*_offset_ [°C]	136.07 ± 0.35	135.04 ± 0.47	163.69 ± 0.55	163.82 ± 0.32	195.89 ± 0.67	196.92 ± 0.34
Δ*H* [J g^−1^]	219.36 ± 0.95	143.92 ± 1.02	136.66 ± 1.11	37.76 ± 1.21	122.02 ± 1.02	44.22 ± 1.32
*T*_offset_–*T*_onset_ [°C]	24.05 ± 0.88	18.58 ± 1.21	12.86 ± 0.98	10.87 ± 0.75	11.90 ± 0.81	10.48 ± 0.43

*T*_onset_—temperature at the beginning of the melting process; *T*_offset_—temperature at the end of the melting process; *T*_offset_–*T*_onset_—difference between temperatures at the beginning and end of the melting process; Δ*H*—melting enthalpy; data are presented as means ± SD.
